# Application of an *Escherichia coli* triple reporter strain for *at‐line* monitoring of single‐cell physiology during L‐phenylalanine production

**DOI:** 10.1002/elsc.202100162

**Published:** 2022-02-26

**Authors:** Manh Dat Hoang, Dieu Thi Doan, Marlen Schmidt, Harald Kranz, Andreas Kremling, Anna‐Lena Heins

**Affiliations:** ^1^ Chair of Biochemical Engineering Department of Energy and Process Engineering TUM School of Engineering and Design Technical University of Munich Garching Germany; ^2^ Systems Biotechnology Department of Energy and Process Engineering TUM School of Engineering and Design Technical University of Munich Garching Germany; ^3^ Gen‐H Genetic Engineering Heidelberg GmbH Heidelberg Germany

**Keywords:** *at‐line* monitoring, *Escherichia coli*, fluorescent reporter strain, L‐phenylalanine, population heterogeneity

## Abstract

Biotechnological production processes are sustainable approaches for the production of biobased components such as amino acids for food and feed industry. Scale‐up from ideal lab‐scale bioreactors to large‐scale processes is often accompanied by loss in productivity. This may be related to population heterogeneities of cells originating from isogenic cultures that arise due to dynamic non‐ideal conditions in the bioreactor. To better understand this phenomenon, deeper insights into single‐cell physiologies in bioprocesses are mandatory before scale‐up. Here, a triple reporter strain (3RP) was developed by chromosomally integrating the fluorescent proteins mEmerald, CyOFP1, and mTagBFP2 into the L‐phenylalanine producing *Escherichia coli* strain FUS4 (pF81_kan_) to allow monitoring of growth, oxygen availability, and general stress response of the single cells. Functionality of the 3RP was confirmed in well‐mixed lab‐scale fed‐batch processes with glycerol as carbon source in comparison to the strain without fluorescent proteins, leading to no difference in process performance. Fluorescence levels could successfully reflect the course of related process state variables, revealed population heterogeneities during the transition between different process phases and potentially subpopulations that exhibit superior process performance. Furthermore, indications were found for noise in gene expression as regulation strategy against environmental perturbation.

**Abbreviations**
L‐phe: L‐phenylalanine
L‐tyr: L‐tyrosine
3RP: triple reporter strain

## 1 INTRODUCTION

Biotechnological production processes are a sustainable alternative to chemical production industries, as microorganisms are capable of producing a great variety of products for the food, feed, and pharmaceutical industries [1]. An example is the production of L‐phenylalanine (L‐phe), an important building block for sweeteners in the food industry, which can be produced by *Escherichia coli* from glycerol [2].

**PRACTICAL APPLICATION**

Genetically encoded fluorescent reporter strains express fluorescent proteins together with cellular events of interest. Triggering of these events can then be monitored via fluorescence measurement. Developing multiple instead of nowadays commonly applied single reporter strains raises these strains as noninvasive tools for bioprocess monitoring to a next level as they enable simultaneous monitoring of different single‐cell characteristics, which can then be correlated to each other and to process performance on population level. This information leads to increased understanding of bioprocess events, heterogeneities, and enlighten cell–cell and cell–bioreactor interactions, which would be masked when solely considering population level physiology. In bioprocesses, potential subpopulations with superior properties for improved productivity can be uncovered. Thus, multiple reporter strains shall support future bioprocess design and supervised up‐scaling by already knowing coexisting phenotypes before potentially experiencing productivity loss due to unspecific population heterogeneities induced by non‐ideal process conditions.

Despite promising concepts, a rather modest number of bioprocesses has resulted in industrial scale production and marketed products [3]. One major reason is that the scale‐up of bioprocesses to production scale often results in lowered yields and productivity compared to the respective well‐mixed lab‐scale processes due to the omnipresent phenomenon population heterogeneity [4, 5]. Although the producing cells in the bioreactor originate from isogenic cultures, the phenotype of single cells can differentiate significantly especially during large‐scale bioprocesses, so that potentially even distinct subpopulations arise [6]. The reason is that fluctuating environmental conditions with gradients in process state variables occur due to mixing insufficiencies and mass transfer limitations in large‐scale bioprocesses. As a consequence, each cell experiences a random order of microenvironments (lifelines) and thus exhibits different single‐cell physiologies matched with its lifeline in the bioreactor. This leads to formation of a heterogeneous culture with potentially deviating single cell productivity [7, 8]. Even though the consequences of population heterogeneity in bioprocesses can nowadays be studied with different available experimental tools, mechanistic understanding of this phenomenon is still comparably low [9].

One prominent approach to get insights on physiology of cells is the utilization of fluorescent reporter strains [10, 11]. These are strains in which fluorescent proteins are integrated into specific operons or loci of interest. Therefore, the triggering event for their expression can be monitored by measurement of fluorescence levels of the reporter strain. These fluorescence levels are almost exclusively measured with flow cytometry as it allows *at‐line* high‐throughput single‐cell data acquisition during cultivations [12, 13]. For the design of reporter strains, a great diversity of fluorescent proteins is available which differ in characteristics such as brightness, maturation times, and oligomeric state [14]. However, mostly fast maturating fluorescent proteins with bright detectability and no cytotoxic effects on the host strain are desirable [13, 14]. There are various single‐reporter strains already available, allowing monitoring of different single‐cell characteristics such as growth, stress responses of different kind and cellular fitness [15, 16, 17, 18]. There are also reporter strains that can sense nutrient or oxygen limitation as well as intracellular stress factors like imbalances in redox state, intracellular pH or accumulation of oxygen radicals [19, 20, 21, 22]. Product formation reporter strains can identify the best producing cells in a bioprocess or detect loss in productivity during scale‐up [23, 24].

More efficient, however, is the application of multiple reporter strains combining reporter molecules for monitoring of several cellular characteristics, whose response can then be directly correlated to each other [25]. This raises the level of understanding of cellular interactions and comprises a powerful alternative tool to omics technologies, which are time‐consuming and not yet capable of providing data on single‐cell level [26].

To the best of our knowledge, only few multiple reporter strains exist [27, 28]. One example is the *E. coli* triple reporter strain (3RP) described by Heins et al. (2020) in which three fluorescent proteins were chromosomally integrated into the *rrnB* operon, the *narGHIJ* operon, and downstream of the *rpoS* gene for monitoring of growth, oxygen availability, and the general stress response of single cells [25]. In the present study, the general reporter strain concept was adapted to generate a 3RP based on the previously well‐characterized L‐phe producing *E. coli* strain FUS4 (pF81_kan_) [29]. The aim was to apply this 3RP in fed‐batch processes for L‐phe production in a well‐mixed stirred‐tank bioreactor at lab‐scale to uncover single‐cell phenomena and potential formation of subpopulations that contribute to process performance of FUS4 (pF81_kan_). A special focus was put on investigating physiological changes in different process phases as, for instance, during the product formation phase, when a decline in product formation coupled to loss in cellular activity was consistently found in previous studies with FUS4 (pF81_kan_) [29].

## 2 MATERIALS AND METHODS

### 2.1 *Escherichia coli* strains

The recombinant *E. coli* strain FUS4 (pF81_kan_) was used for cultivation in the L‐phe production process [29, 30]. FUS4 is a derivative of *E. coli* K‐12 with deletion of chromosomal genes *pheA*, *aroF*, and *tyrA* along the aromatic biosynthesis pathway. Consequently, cells are auxotroph toward L‐phe and L‐tyrosine (L‐tyr). FUS4 harbors the pF81_kan_ plasmid encoding for the genes *aroF*, *pheA*, *aroB*, and *aroL* under the control of an inducible *P_tac_* promoter system. This allows overexpression of deleted enzymes along the aromatic biosynthesis pathway for production of L‐phe. The plasmid further provides kanamycin resistance, which can be used as selection marker [29]. In this study, FUS4 (pF81_kan_) was transformed into a 3RP by a series of knock‐in recombination reactions for site‐specific insertion of three synthetic cassettes using λ‐red recombination and a subsequent FLP/FRT mediated recombination reaction [31]. Each cassette carried a monocopy of the coding sequence (CDS) of a fluorescent protein. A synthetic copy of mEmerald was inserted into the ribosomal *rrnB* promoter complex with a synthetic ribosomal binding site (RBS) 5′‐AAAGAGGAGAAA‐3′ according to Elowitz and Leibler (2000) and a transcriptional terminator downstream of the CDS [32]. This cassette was integrated in the rhamnose operon with simultaneous deletion of the native genes *rhaB* and *rhaS*. Subsequently, the mTagBFP2 gene was inserted downstream of the *rpoS* gene in conjunction with its own RBS. Last, the CyOFP1 gene was inserted into the *nar*GHIJ gene cluster downstream of all native genes together with the synthetic RBS mentioned above. These insertions allow to follow single‐cell growth, oxygen limitation, and general stress response of single cells by fluorescence of *rrnB*‐mEmerald, *rpoS*‐mTagBFP2, and *nar*‐CyOFP1, respectively [25].

### 2.2 Preliminary culture preparation

Cryopreserved cells of *E. coli* FUS4 (pF81_kan_) and *E. coli* 3RP (pF81_kan_) were streaked on minimal medium agar plates with glycerol as carbon source prepared according to Weiner et al. (2014) [29] and incubated at 37°C for at least 66 h. One single colony was then used for inoculation of a 100 mL shake flask with 10 mL minimal medium with 7 g/L of glycerol as carbon source prepared according to the same protocol as the agar plates [29]. After cultivation at 37°C and 150 rpm for approximately 24 h in an orbital shaker (Multitron, Infors HT, Switzerland), the optical density at 600 nm (OD_600_) was measured (Genesys 10UV, Thermo Fisher Scientific, USA). A defined volume of cell suspension was transferred to two 500 mL shake flasks each one with 100 mL minimal medium to yield a starting OD_600_ of 0.01. These cultures were further cultivated at 37°C and 250 rpm for at least 24 h. When the cells reached exponential growth phase with an OD_600_ above 0.5, the cultures were centrifuged at 3260×*g* for 10 min at 4°C. The supernatant was discarded and the cell pellets were suspended in fresh minimal medium. Bioreactor cultivations were inoculated with washed cells to a starting OD_600_ of 0.1.

### 2.3 Bioreactor cultivation

Fed‐batch processes for L‐phe production with *E. coli* FUS4 (pF81_kan_) and *E. coli* 3RP (pF81_kan_) on minimal medium with 4 g/L glycerol as sole carbon source, as described by Weiner et al. (2014) [29], were conducted in a 3.6 L stirred‐tank bioreactor (Labfors 5, Infors GmbH, Germany), which was equipped with three baffles and two six‐blade flat‐blade turbines. Prior to inoculation, the medium was pumped into the bioreactor under sterile conditions to a starting volume of 1 L. During the process, temperature was kept at 37°C and a pH electrode (EasyFerm Plus PHI Arc 325, Hamilton, USA) was implemented for pH control at 7.0 ± 0.1 with 42% phosphoric acid and 25% ammonia. Dissolved oxygen (pO_2_) levels were monitored by a pO_2_ probe (VisiFerm DO Arc 325 H0, Hamilton, USA) and were maintained above 30% by the stepwise increase of either stirrer speed (maximum: 1500 rpm) or aeration rate (maximum: 5 L/min). Both sensors were calibrated according to standard procedures using a two‐, respectively one‐point calibration (pH 4.0 and 7.0, calibration for 100% pO_2_). An antifoam probe allowed the controlled addition of antifoam solution (AF204, Sigma–Aldrich, USA) to circumvent over foam reactions. Online analysis of off‐gas oxygen (O_2_) and carbon dioxide (CO_2_) was performed using a gas sensor (BlueVary, BlueSens, Germany).

The process strategy was adapted from Weiner et al. (2014) [29]. The fed‐batch process for L‐phe production can be divided into three distinctive phases: a batch phase, a biomass production phase, and a product formation phase. After the batch phase, whose end was characterized by glycerol depletion and recognized by a steep increase in pO_2_ levels in the bioreactor, the biomass production phase was started. In this phase, an exponential feed with a growth rate of *μ*_set_ = 0.1 h^–1^ was applied with two consecutive feed media in which the second feed medium was applied after the first feed medium was empty. Feed medium one contained 120 g/L glycerol, 2.5 g/L L‐phe, 3.6 g/L L‐tyr, 60 g/L ammonium sulfate, and 0.1 g/L kanamycin, whereas feed medium two consisted of 400 g/L glycerol, 1.11 g/L L‐phe, 3.8 g/L L‐tyr, 25 g/L ammonium sulfate, and 0.1 g/L kanamycin. Both media were titrated with either 25% ammonia or 5 M potassium hydroxide to allow the complete dissolving of L‐tyr. Provision of a biomass concentration of at least 20 g/L indicated the transition to the product formation phase in which the cells were induced with 0.3 mM IPTG. Additionally, feed medium three was constantly applied with a rate of 0.18 g_glycerol_/g _biomass_/h, which contained 800 g/L glycerol, 8 g/L ammonium sulfate, 8 g/L ammonium phosphate, and 0.1 g/L kanamycin. At the start of supply of feed medium one and two, 4.8 or 9.6 mL of minimal media without amino acids and glycerol was added whereas at the start of supply of feed medium three, 8.8 mL of a four times concentrated minimal media solution without amino acids and glycerol was injected to the cultivation broth [29]. Samples for high‐performance liquid chromatography (HPLC), cell dry weight measurements and flow cytometry analysis were withdrawn frequently during all process phases.

### 2.4 Sample analysis

For measuring the cell biomass, empty 2 mL centrifuge tubes were dried at 80°C for at least 24 h and weighted. After collecting 2 mL cell sample, they were centrifuged at 21,130×*g* and 4°C for 20 min. The cell pellet was dried at 80°C for at least 24 h. The weight difference between dried empty tubes and tubes with cell pellets divided by the volume revealed the biomass concentration.

Samples for the quantification of extracellular metabolite concentrations were prepared by filtration (pore size 0.2 μm) of the supernatant from dry cell weight measurements and stored at 4°C until analysis.

L‐phe and L‐tyr concentrations were analyzed using a Smartline HPLC (Knauer, Germany) coupled to a derivatization protocol with 0.04 M bicine (pH 10.2 titrated with sodium hydroxide) as buffer solution as described by Weiner et al. (2014) but with higher sample volumes of 11 μL to enhance detectability [29].

Organic metabolites such as glycerol, acetate, and lactate were quantified by the Prominence‐I LC‐2030C HPLC (Shimadzu, Japan) equipped with an ion‐exchange column (Aminex HPX‐87H 300 mm × 7.8 mm, Bio‐Rad, USA). An isocratic flow of 0.6 mL/min of 5 mM sulfuric acid and a constant temperature of 60°C were applied during separation. 10 μL of sample were injected and the quantification of the components was done with a RID‐20A refractive index detector (Shimadzu, Japan).

Samples for flow cytometry analysis of fluorescence were prepared by centrifugation at 21,130 × g and 20°C for 3 min. The cell pellets were suspended in phosphate saline buffer (0.2 g/L potassium chloride, 0.24 g/L mono‐potassium phosphate, 8 g/L sodium chloride, and 1.44 g/L di‐sodium phosphate). Fluorescence of the cells at different process stages was measured with a FACSMelody (BD, USA). This device is equipped with three lasers allowing excitation at 405 nm (36 mW), 488 nm (16 mW), and 640 nm (36 mW) and nine detection filters. A sorting nozzle with a diameter of 100 μm was applied. As sheath fluid, FACSFlow (BD, USA) was used and measurement was done with a rate of 1000 events per second recording 100,000 events. Background noise signals were circumvented by application of a threshold on the side scatter (SSC). Photon multiplier tube voltages for forward scatter (FCS) and SSC as well as the detection filters 448/45 nm for mTagBFP2 (excited by the 405 nm laser), 527/32 nm for mEmerald, and 586/42 nm for CyOFP1 fluorescence (both excited by the 488 nm laser) were set to 250, 335, 500, 500, and 600 mV, respectively. Distinct signal detection of mTagBFP2, mEmerald, and CyOFP1 fluorescence without overlap was confirmed in preliminary experiments (see Supplementary material Figures S1–S3).

Autofluorescence measurements were done measuring fluorescence of the reference strain *E. coli* FUS4 (pF81_kan_) in the above‐mentioned filters, which allowed detection of mTagBFP2, mEmerald, and CyOFP1 fluorescence.

### 2.5 Data analysis

Fluorescence measurements were conducted based on the pulse area and saved with FACSChorus (BD, USA), and the raw data were exported as FCS 3.1 files. Data analysis was conducted with FCS Express 7 (De Novo Software, USA) and Matlab (Mathworks, USA). This includes the calculation of median, skewness, and coefficient of variance (CV) of fluorescence distributions for the three reporter proteins. The latter was calculated by dividing the standard deviation of the distribution by its mean and is a measure of the level of population heterogeneity. Pearson's first coefficient of skewness is calculated by dividing the difference between the mean and mode of the distribution by its standard deviation. Since distributions are shown in logarithmic scale, all distributions are right‐skewed; however, a decrease in skew might indicate left‐skewed distributions. Stacked offset histogram plots were generated visualizing changes in fluorescence distributions during the L‐phe production process. Furthermore, density plots were created for chosen process time points (15, 37, 43, and 94 h), correlating fluorescence at 448/45 nm versus 527/32 nm, 586/42 nm versus 527/32 nm, and 448/45 nm versus 586/42 nm. When fluorescence distributions exhibited distinct subpopulations, a gate was set corresponding to the local minimum between the subpopulations. Then, the percentage of the population that is found in both subpopulations was calculated.

## 3 RESULTS

### 3.1 Influence of genomic engineering on process performance

Since genomic integration of several fluorescent proteins into a production host potentially results in metabolic burden, process performance of the triple reporter in fed‐batch cultivations for L‐phe production was evaluated based on population level physiology in comparison to the reference strain without fluorescent proteins. Both, the reference strain *E. coli* FUS4 (pF81_kan_) and the modified strain *E. coli* 3RP (pF81_kan_), were cultivated in a well‐mixed stirred‐tank bioreactor at lab‐scale for L‐phe production. The process was adapted from Weiner et al. (2014) and consisted of three phases including a batch phase, followed by a biomass production phase and finally a product formation phase in which the cells were induced with 0.3 mM IPTG [29].

**FIGURE 1:**
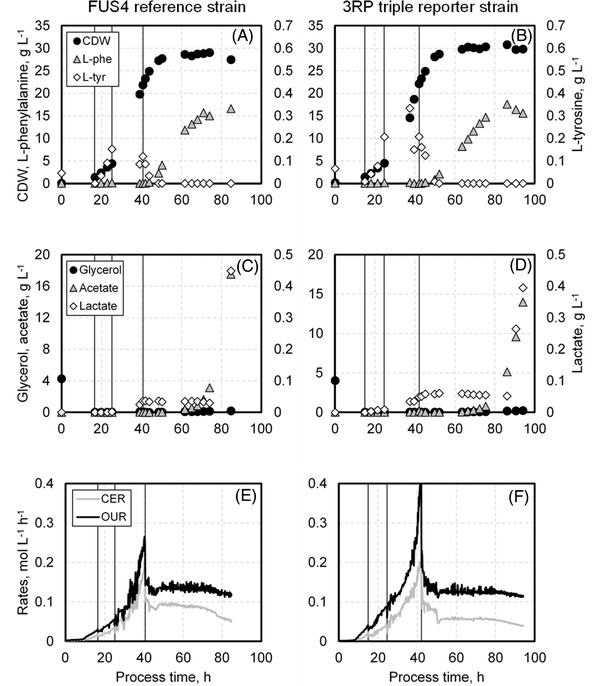
Fed‐batch process for L‐phenylalanine production with *Escherichia coli* FUS4 (pF81_kan_) (A, C, E) and the triple reporter strain *E. coli* 3RP (pF81_kan_) (B, D, F) in a 3.6 L stirred tank bioreactor. A and B show concentrations of biomass (black circles), L‐phenylalanine (gray triangles) and L‐tyrosine (white diamonds) in the course of the bioprocess, whereas C and D display concentrations of the substrate glycerol (black circles) and the by‐products acetate (gray triangles) and lactate (white diamonds). E and F provide the oxygen uptake (OUR, black line) and carbon emission rates (CER, gray line). The first vertical line in each graph indicates the start of the biomass production phase (16 h or 15 h) after a batch phase, while the second vertical line marks the switch to higher concentrated feed media at 25 h. Product formation phase (third vertical at 40 h or 42 h) starts upon induction with 0.3 mM IPTG. Both strains were cultivated using minimal medium with glycerol as carbon source. CER, carbon emission rate; OUR, oxygen uptake rate; 3RP, triple reporter strain

After the initial batch phase of around 15.0 h to 16.0 h, both *E. coli* FUS4 (pF81_kan_) and *E. coli* 3RP (pF81_kan_) showed a linear increase of biomass in the subsequent feeding phase until 40.7 h respectively 42.2 h of process time (Figure 1A and B). During that phase, concentrations of the auxotrophic amino acids, L‐phe and L‐tyr, were below 0.4 g/L. The stronger slope of biomass increase at the end of this phase is due to the application of a differently concentrated feeding solution from 25.2 h onwards. With provision of a sufficiently high biomass concentration of over 20 g/L after 40.7 h respectively 42.2 h of process, the cells were induced with 0.3 mM IPTG marking the start of the product formation phase (Figure 1, second vertical line). Eight hours later, L‐tyr was fully depleted and the biomass concentration remained level at a maximum of 29.05 ± 0.43 g/L for *E. coli* FUS4 (pF81_kan_) and 30.78 ± 0.75 g/L for *E. coli* 3RP (pF81_kan_), respectively. Simultaneously, both strains started to produce L‐phe and achieved a maximum product concentration of 16.6 g/L and 17.6 g/L at the end of the cultivation with *E. coli* FUS4 (pF81_kan_) and *E. coli* 3RP (pF81_kan_), respectively. After 70 h, respectively 74 h of process, product formation declined in both strains accompanied by a preceding accumulation of by‐products such as lactate and acetate (Figure 1C and D) that were not produced in earlier process stages. Nevertheless, glycerol as sole carbon source was still fully consumed by the cells (Figure 1C and D).

The oxygen uptake rate (OUR) and carbon emission rate (CER) (Figure 1E and F) increased during the biomass production phase, reaching their highest values between 40 h to 42 h of process. Both decreased after induction and remained level after product formation started. With declining product formation, their levels gradually decreased further until the end of the process for both strains.

### 3.2 *At‐line* monitoring of cellular characteristics by averaged single‐cell fluorescence

Next, median fluorescence values of distributions for growth, oxygen availability, and general stress response of single cells (*rrnB*‐mEmerald, *narGHIJ‐*CyOFP1, and *rpoS*‐mTagBFP2, respectively) were correlated with the growth rate on population level, the dissolved oxygen level in the bioreactor, and the product formation rate during the L‐phe production process, respectively.

**FIGURE 2:**
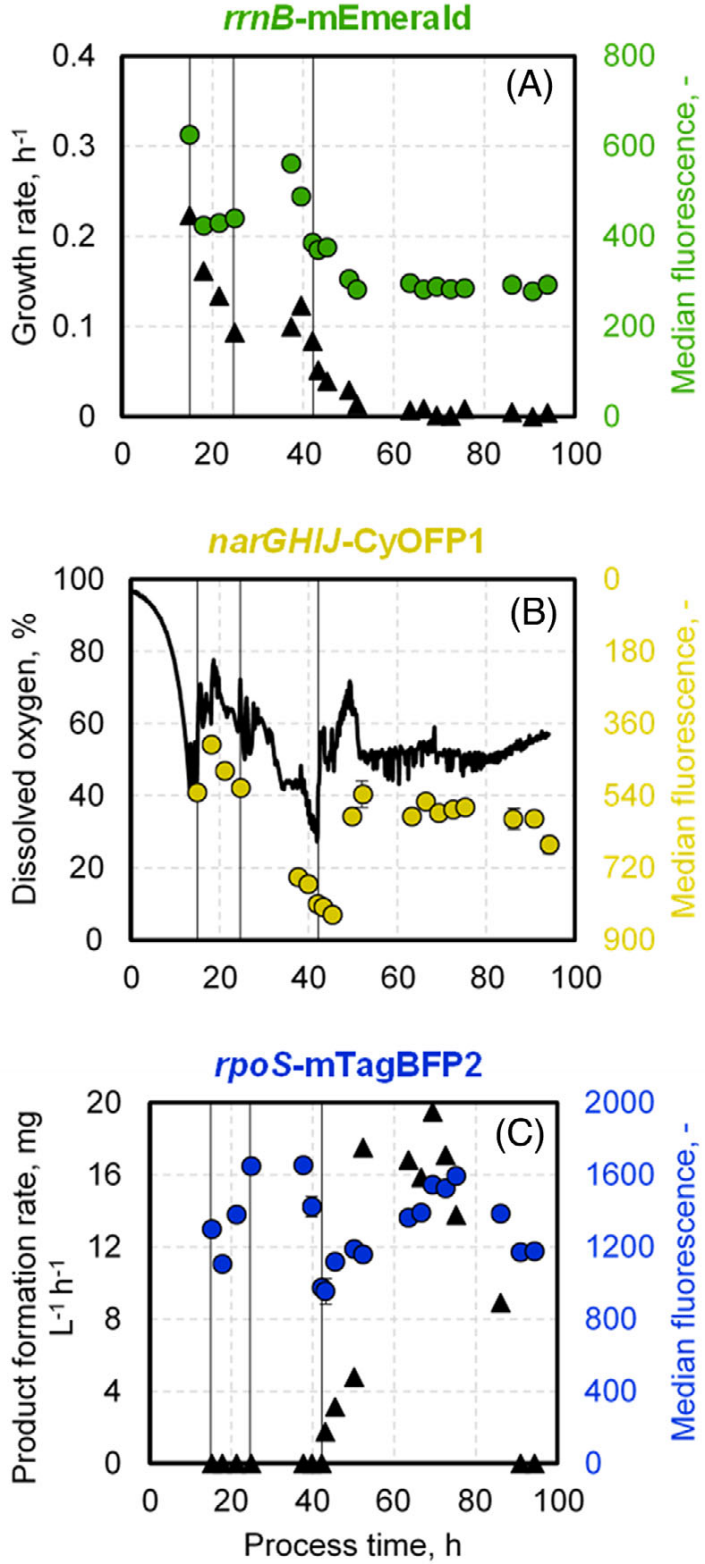
Median fluorescence measurements during the L‐phenylalanine production process with *E. coli* triple reporter strain (3RP) (pF81_kan_) in a 3.6 L stirred tank bioreactor. Median fluorescence of mEmerald is coupled to the expression of the *rrnB*‐operon for monitoring of single cell growth and is therefore plotted together with the growth rate on population level (A). B shows the dissolved oxygen levels in the bioreactor together with median fluorescence of *narGHIJ‐*CyOFP1 as marker for oxygen availability. mTagBFP2 is coupled to the expression of the *rpoS* gene and provides a correlation to general stress response levels. These data are plotted together with the biomass specific product formation rate (C). The first vertical line in each graph indicates the start of the biomass production phase (15 h) after a batch phase, while the second vertical line marks the switch to higher concentrated feed media at 25 h. Product formation phase (third vertical at 42 h) starts upon induction with 0.3 mM IPTG. The strain was cultivated using minimal medium with glycerol as carbon source

Following the growth rate of 3RP on population level (Figure 2A), three distinctive levels were visible. The highest growth rate of 0.22 h^–1^ was achieved at the end of the initial batch phase at around 15 h of process time. Afterwards, during the biomass production phase, the growth rate decreased to around 0.1 h^–1^ due to the controlled feeding strategy. In the product formation phase, the growth rates further declined to almost no growth after 60 h of process time. Expectedly, these three growth levels were mirrored by the corresponding median fluorescence of mEmerald. The highest median fluorescence level of 624 ± 5.2 was reached at the end of the initial batch phase, while an intermediate median fluorescence of 454.8 ± 61.7 was monitored during the biomass production phase. During the product formation phase, median fluorescence further decreased within 10 h; thereafter, it only gradually declined further until reaching a value below 300 at the end of the process.

With the increase of biomass during the biomass production phase, dissolved oxygen levels in the bioreactor continuously decreased from approximately 75% to 30% (Figure 2B). From 18 h of process time onward, which correlated with a dissolved oxygen level in the bioreactor of around 70%, the median fluorescence of CyOFP1 correlated to oxygen limitation constantly increased from 412.4 ± 6.1 to 821.1 ± 13.9, displaying its peak values at the end of the biomass production phase and the beginning of the product formation phase, respectively. With the induction of product formation at around 42 h of process time, the dissolved oxygen level rose after a short delay of 1–2 h and remained level at around 50%. Simultaneously, the median fluorescence dropped before staying constant at 577.5 ± 20.9.

Median fluorescence values of mTagBFP2 coupled to expression of the *rpoS* gene for *at‐line* monitoring of the general stress response of single cells showed two peaks during the L‐phe production process (Figure 2C). In the biomass production phase, an increase of median fluorescence from 1107.2 ± 1.7 at 18 h to its highest value at 1649.2 ± 14.8 at 24.6 h was detected. Afterwards, the median remained about level before starting to decline 5 h before induction of the cells. With induction at around 42 h of process, median fluorescence levels were lowest at 965.5 ± 14.5. Afterwards, they constantly increased reaching a maximum of 1593.8 ± 14.2 after around 75 h of process, before declining again until the process was stopped. In comparison with the biomass specific product formation rate on population level, it is interesting that after 52 h of process time, the cells reached their highest product formation rate of 16.8 ± 1.9 mg_L‐phe_/g _biomass_/h, which remained constant until 75 h of process when the rate started to decrease. Consequently, the highest general stress response levels coincided with the start of product formation decline.

### 3.3 Single‐cell fluorescence distribution during the L‐phenylalanine production process

**FIGURE 3:**
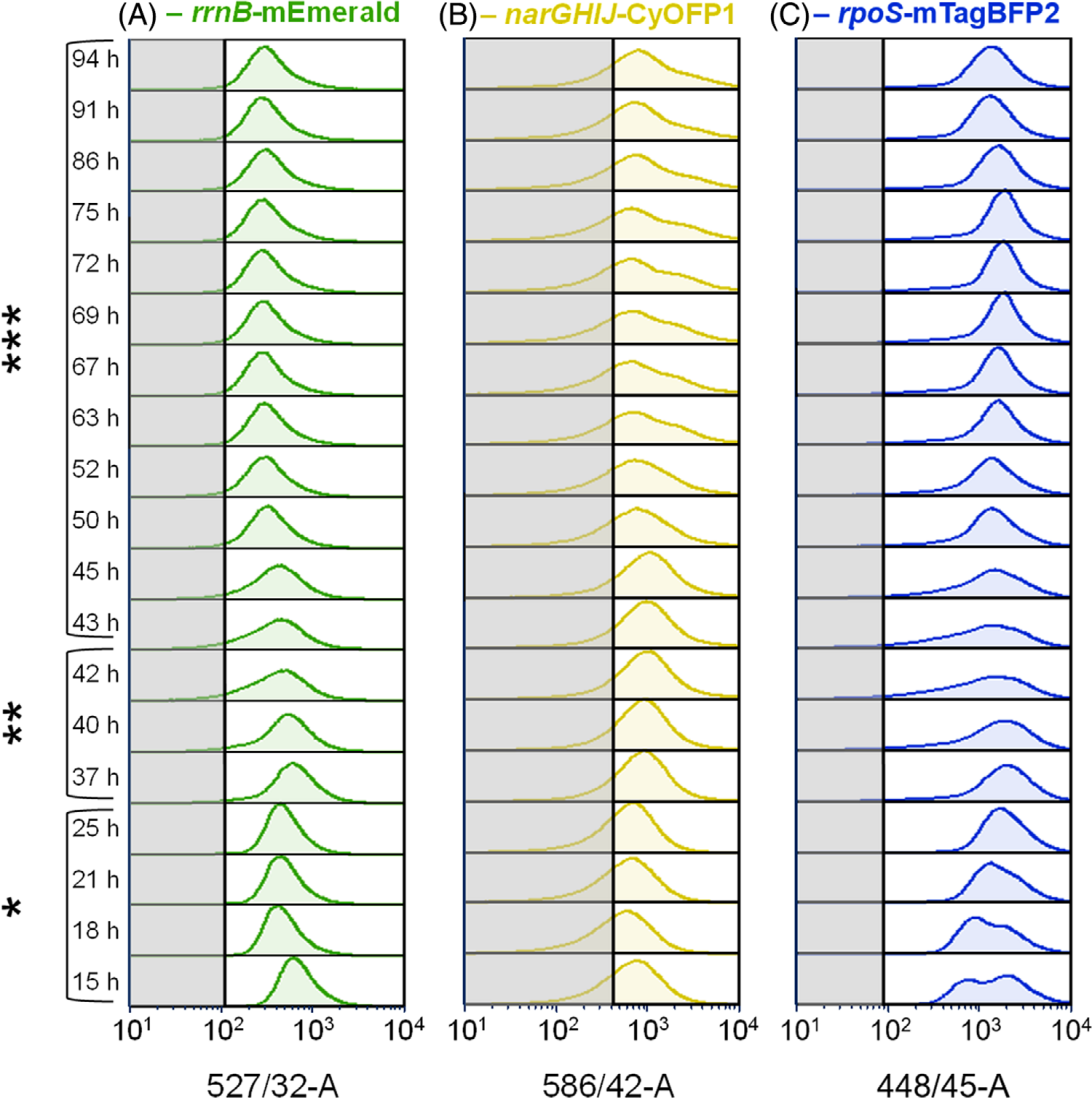
Fluorescence distributions as cell count against fluorescence intensity for the L‐phenylalanine production process with *E. coli* triple reporter strain (3RP) (pF81_kan_) in a 3.6 L stirred tank bioreactor. Stacked histograms show fluorescence distributions for *rrnB‐*mEmerald (A), *narGHIJ*‐CyOFP1 (B), and *rpoS‐*mTagBFP2 (C) following the bioprocess. The gray area indicates the maximum of autofluorescence distributions during the cultivation of the *E. coli* FUS4 (pF81_kan_) reference strain. The time points and phase of the process the distributions originate from are depicted on the left side of the graphs. The strain was cultivated using minimal medium with glycerol as carbon source. *Biomass production phase with feed media 1, **biomass production phase with feed media 2, and ***product formation phase

**FIGURE 4:**
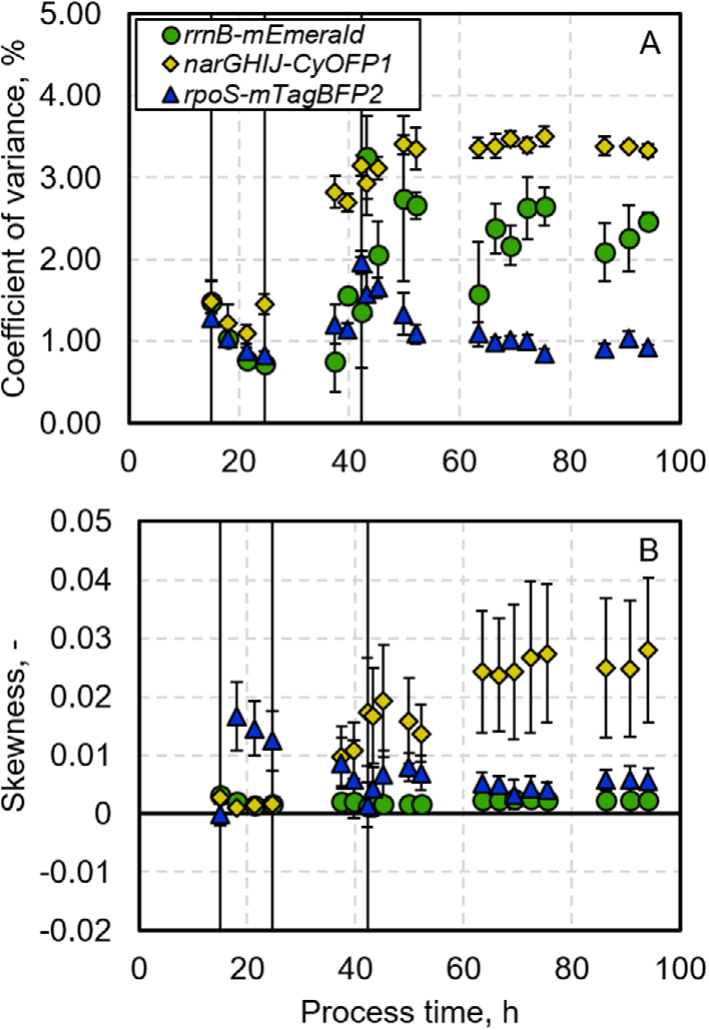
Skewness (A) and coefficient of variance (B) for fluorescence distributions of *rrnB‐*mEmerald, *narGHIJ*‐CyOFP1, and *rpoS‐*mTagBFP2 at 527/32‐A, 586/42‐A during the L‐phenylalanine production process with *E. coli* 3RP (pF81_kan_) in a 3.6 L stirred tank bioreactor. The first vertical line in each graph indicates the start of the biomass production phase (15 h) after a batch phase, while the second vertical line marks the switch to higher concentrated feed media at 25 h. Product formation phase (third vertical at 42 h) starts upon induction with 0.3 mM IPTG. The strain was cultivated using minimal medium with glycerol as carbon source

To consider potential unequal behavior of single cells, histogram distributions plots for growth, oxygen limitation, and general stress response following the L‐phe production process were generated (Figure 3) as well as skewness and CV for the respective distributions plotted (Figure 4). Additionally, respective autofluorescence measurements, taken during the L‐phe production process with *E. coli* FUS4 (pF81_kan_), are depicted.

Single cell growth (*rrnB*‐mEmerald) followed a mono‐modal distribution until 37 h of process in the biomass production phase (Figure 3A) with a low constant CV value of 0.96 ± 0.32 (Figure 4A). Between 37 and 45 h of the process, during the transition from biomass production phase to product formation phase, distributions broadened with tailing toward lower fluorescence intensities, which resulted in declining skew and exponentially increasing CV values (Figure 4). The effect enhanced the longer the transition period lasted. From 50 h onwards, distributions narrowed again at lower fluorescence intensity accompanied by decreasing CV values (Figure 4A). Concurrently, tailing toward higher fluorescence intensities (Figure 3A) was confirmed by increased skew (Figure 4B). Moreover, toward the end of the process, gradual broadening can be suspected. Median autofluorescence was low with average value at 111.3 ± 19.2 and thus only marginally overlapping with fluorescence signals of *E. coli* 3RP (pF81_kan_) (Figure 3A).

The overlap between autofluorescence signals and CyOFP1 fluorescence distributions of *E. coli* 3RP (pF81_kan_) were more pronounced (average median autofluorescence: 423.2 ± 70.0; Figure 3B). However, the main part, covering 71.8% ± 6.3%, of oxygen limitation distributions (*narGHIJ*‐CyOFP1) of *E. coli* 3RP (pF81_kan_) settled at higher fluorescence intensities compared to autofluorescence values and exhibited mono‐modal distributions with relatively low CV values (Figure 4A) until 45 h of process. They tailed toward lower fluorescence intensities, which got less pronounced with time, however. From 52 h of process until the process was stopped, distributions significantly increased in skew (Figure 4B) and reached almost double width, compared to distributions at the end of the batch phase, which caused an increase in CV values (Figure 4A). Starting from 63 h of process, a small higher fluorescent subpopulation of 22.6% adjacent to the main population appeared that increased in significance until 75 h of process whereon it gradually declined again.

General stress response levels of single cells (*rpoS*‐mTagBFP2) revealed more distinct changes following the L‐phe production process (Figure 3C). At the end of the batch phase, at 15–18 h of process, two adjacent distributions were visible covering 40%–60% of cells in the higher fluorescent subpopulation. However, the distribution changed into a mono‐modal distribution within 6 h, which caused a decrease in CV values (Figure 4A). Afterwards, distributions broadened with increased tailing toward lower fluorescence intensities, which decreased their skew and enhanced their CV values (Figure 4), during the transition from biomass production to product formation phase between 37 and 45 h of process. After 50 h, the distributions slowly got less skewed (Figure 4B) und more uniform (Figure 4A) at higher fluorescence intensities until 75 h of process and then gradually broadened again. The average median autofluorescence was at 88.7 ± 36.2 and thus marginally overlapping with fluorescence levels of *E. coli* 3RP (pF81_kan_) (Figure 3C).

### 3.4 Correlation between the fluorescent markers of the triple reporter strain

**FIGURE 5:**
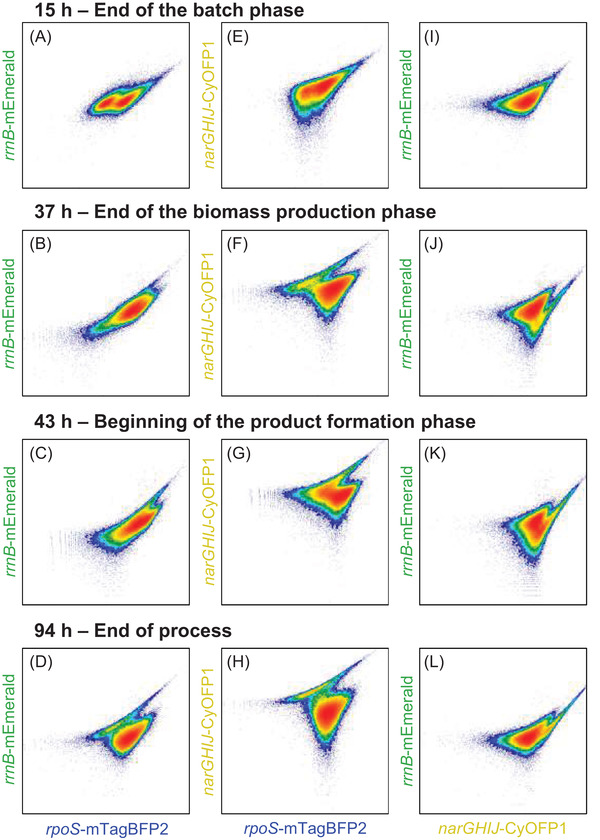
Density plots of combinations of fluorescence intensities at 15 (end of the batch phase), 37 (end of the biomass production phase), 43 (start of the product formation phase), and 94 h (end of the process) during the L‐phenylalanine production process with *E. coli* triple reporter strain (3RP) (pF81_kan_) in a 3.6L stirred tank bioreactor. A–D show the fluorescence intensity at 448/45‐A versus 527/32‐A, while E–H show the correlation between 448/45‐A and 586/42‐A. I–L show fluorescence data of 586/42‐A versus 527/32‐A. Fluorescence ranges of 527/32, 586/42, and 448/45 nm allow detection of *rrnB*‐mEmerald (growth), *narGHIJ*‐CyOFP1 (oxygen availability), or *rpoS*‐mTagBFP2 (general stress response), respectively. The strain was cultivated using minimal medium with glycerol as carbon source. Induction was done by the addition of 0.3 mM IPTG

When applying multiple instead of single reporter strains, distributions of different fluorescent markers can be pairwise correlated with each other in biplots, allowing deeper insights into their interconnection (Figure 5). As fluorescence distributions, (Figure 3), skewness, and CV values (Figure 4) indicated the significant changes in single‐cell fluorescence at the end of the batch phase (15 h), during the transition from biomass production phase to product formation phase (37–43 h) and at the end of the process (94 h), a focus was set on these process stages.

Correlating general stress responses to the single‐cell growth showed two adjacent populations exhibiting two different levels of general stress response; however, the same growth characteristics were seen at 15 h of process. These, however, transformed into a uniform population during the transition from the biomass production phase (37 h) to the product formation phase (43 h). At the end of the process, two populations were visible, one showing higher mEmerald levels (10%) than the majority of the cells (Figure 5A–D).

When plotting the single‐cell oxygen availability in relation to the general stress responses, one population appeared at the end of the batch phase with a slight difference in general stress response levels within the population. Interestingly, a minority of cells (13%) showed increased levels of oxygen limitation at the end of the biomass production phase at 37 h. This subpopulation even increased to 26.5% at the beginning of the product formation phase at 43 h but shrank again to 11% at the end of the process. Some cells of this subpopulation did not only show higher oxygen limitation levels, but also lower general stress response levels (Figure 5E–H).

The correlation of single‐cell oxygen availability with single‐cell growth revealed a uniform distribution at 15 h of process. This population broadened over time, with a small subpopulation in the oxygen availability marker with gradually increasing fluorescence intensity until the end of the process. Furthermore, this subpopulation exhibited weaker growth characteristics than the main population (Figure 5I–L).

## 4 DISCUSSION

Reporter strains have been proven to be useful noninvasive tools to monitor cellular characteristics in bioprocesses in real‐time [33, 34, 35]. In the present study, the previously well‐characterized L‐phe producing strain *E. coli* FUS4 (pF81_kan_) was transformed into a 3RP by chromosomally integrating the fluorescent proteins, mEmerald, CyOFP1, and mTagBFP2, so that they are expressed together with the ribosomal promoter *rrnB*, the *narGHIJ*‐operon, and the alternative sigma factor *rpoS*, respectively. The resulting strain was successfully applied to monitor growth, oxygen limitation, and general stress responses of singles cells in the L‐phe production processes in a well‐mixed lab‐scale bioreactor. General functionality of the 3RP concept was already demonstrated in an earlier study [25], but the fluorescent proteins for monitoring oxygen availability and general stress response, TagBFP657 respectively mStrawberry, were exchanged by CyOFP1 and mTagBFP2 in the present strain. Furthermore, applicability of the concept for a strain that is already metabolically challenged by production of L‐phe was ambiguous. Comparing key process performance parameters, such as maximum biomass and product concentrations and growth behavior during the L‐phe production processes of the newly generated *E. coli* 3RP (pF81_kan_) with that of the reference strain *E. coli* FUS4 (pF81_kan_) without fluorescent proteins, no significant impact on process performance was found. Consequently, metabolic burden by expression of the fluorescent proteins next to L‐phe production was obviated. This was expected, as in previous investigations with *E. coli* it was shown that disruption of a considerable portion of genes did not affect cell viability and metabolism [36, 37, 38, 39, 40].

Furthermore, the changes in distributions and median fluorescence signals of the 3RP in bioprocesses for L‐phe production related to cellular growth, oxygen limitation, and general stress response matched the respective timely course of process state variables with a maximum signal delay of 1 h to 2 h.

Monitoring of cell growth was realized by integration of mEmerald into the *rrnB* operon, decoding for a RNA polymerase subunit. Growing cells exhibit higher concentrations of ribosomes, which should trigger *rrnB* expression [41]. Therefore, higher growth rates should lead to higher fluorescence intensities. Indeed, considering the median fluorescence levels of mEmerald, the growth rate on population level was closely reflected in all process phases.

CyOFP1 was integrated into the *nar*GHIJ operon decoding for a nitrate reductase. Whenever cells experience dissolved oxygen levels below 40% in the bioreactor or are surrounded by high nitrate concentrations, the *nar*GHIJ operon is active [20, 42]. Consequently, an inverse correlation between oxygen availability and median CyOFP1 fluorescence was expected. This could be confirmed especially during the biomass production phase in which dissolved oxygen levels decreased with increasing biomass concentration as well as at the beginning of the product formation phase.

mTagBFP2 was integrated downstream to the promoter controlling the expression of the alpha subunit of the alternative sigma factor *rpoS*. This gene is generally known as global stress response factor. Triggering this gene leads to the expression of *rpoS*‐dependent genes decoding for more specific stress response pathways [43]. As a consequence, general stress response levels can be reflected but the underlying reasons remain unclear. What is well known as a strong trigger for the general stress response is the lack of nutrients leading to starvation [44, 45, 46]. Indeed, elevated general stress response levels via blue fluorescence were visible during the biomass production phase. Though substrate was fed at this stage of the process, fed‐batch processes normally provide a minimum of substrate which is immediately consumed by the cells for control of the cell's growth rate [47]. This uncertainty of nutrient availability however could potentially induce the cellular stress response. Besides nutrient availability, the *rpoS* gene is induced by various other suboptimal conditions such as high or low pH values or temperatures and accumulation of toxic metabolites [43]. Especially, the latter is often a major factor when it comes to production decline in recombinant strains [49, 50]. This might supposingly have happened in this study, as the highest blue fluorescence was seen were the L‐phe concentration reached its maximum. Afterwards, general stress response levels decreased simultaneously with the starting decline of product concentration. Consequently, the decline in L‐phe production might be related to the metabolic overload of the cells, which could have been ultimately triggered by accumulation of toxic byproducts. Hence, the measurement of general stress response was interesting; however, for a deeper investigation of the stress response, future reporter strains shall be equipped with fluorescence proteins coupled to more specific stress responses.

The selection of appropriate fluorescent proteins enables distinctive detection of their fluorescence. Even though there are plenty of selectable characteristics [14, 50, 51], choices were made focusing on high brightness for robust detection even at low concentration of the fluorescent protein and low maturation times to provide a rapid response to triggering events. Although the delay between expression and complete maturation of the fluorescent proteins might be a drawback, it could be neglected in the present study as the maturation times of mEmerald, CyOFP1, and mTagBFP2 were all shown to be <20 min in previous studies [14, 52–54] and thus low compared to overall process duration. The in vivo degradation characteristics of the fluorescent proteins are unknown. Very few related techniques are described in the literature [55, 56], which shall be used for upcoming research. One possibility to control degradation is the implementation of degradation tags on the fluorescent protein, which are recognized by the intracellular protease systems such as ClpXP and Lon [57, 58, 59]. With degradation tag sequences, which differ regarding their degradation time, the lifetime of each fluorescent protein could be determined. Nevertheless, it is important to adapt the degradation times to measuring time to avoid complete loss of signal. Despite the uncertainty of in vivo degradation of the reporter molecules, the median fluorescence data still followed the changes in process state variables almost synchronously. Indeed, it is necessary to mention that autofluorescence for CyOFP1 was only marginally lower than the fluorescence intensities measured for the 3RP. This might be related to weak expression levels of the *nar*‐operon in general. Similar findings were also described by Heins et al. (2020), but a slower maturating fluorescent protein was used [25]. Therefore, substitution of the *narGHIJ*‐operon as reporter for oxygen limitation might be advisable.

The reason to apply a 3RP in the L‐phe production process was to uncover single‐cell behavior that is masked when only considering population level physiology. This includes the appearance of subpopulations, quantification of population heterogeneity in different process phases as well as characterization of single‐cell behavior during declining product formation, which was consistently seen in earlier studies, but it could not be explained [29].

Whereas single cells exhibited low uniform levels of oxygen limitation, two temporary subpopulations were seen for the general stress response at the beginning of the process with similar single‐cell growth characteristics. These might have arisen because part of the population in the bioreactor took longer time to adapt to the fed‐batch mode and exhibited a distinct general stress response to reactivate, whereas the remaining part of the population was still prepared to grow. Consistently, earlier studies revealed that cells in stationary phase exhibited different response times to the addition of fresh medium [60]. When the second feed was applied in the biomass production phase, population heterogeneity levels rose notably by strong dispersion of distributions and some cells appeared to be less vital and stronger metabolically challenged by the higher concentrated feed. The reason was possibly a nutrient overload and the subsequent accumulation of metabolites. This finding clearly extends the information gained by just considering population level physiology, revealing that the cells exhibit a diverse reaction to potentially harmful changes in their environment. This might not have been expected, as on population level, no change in cellular physiology was seen in this process phase. In future studies, cells from opposite sites of the distributions should be deeper functionally characterized to uncover the underlying reason for this strong dispersion.

During the transition from biomass production phase to product formation phase, this effect first persisted, as the heterogeneity level was high for single‐cell growth, with the majority of cells first maintaining their growth level even though the culture was induced to start production formation and stop growing. Concurrently, the cells exhibited broader general stress responses. This phenomenon might be explainable by a bet‐hedging strategy to cope with the transition. Bet‐hedging describes the development of coexisting phenotypes within one population, of which some may be currently disadvantageous but prepare these cells for the future environments they might encounter [35, 61, 62].

Following product formation phase, single cell growth and general stress response levels got more uniform, probably because the overload in metabolism, which lowered the vitality of some cells, could be released by‐product formation. However, some more actively growing cells with high stress response levels seemed to exist, which raises the question whether these were able to simultaneously grow and produce. Furthermore, some cells seemed to be stronger challenged during L‐phe production. They exhibited a less effective general stress response, together with higher oxygen limitation levels and lower growth levels. The cells probably tried to fight accumulation of potentially toxic by‐products, which were seen on population level, by using alternative parts of the metabolism [37, 38]. Another strategy the cells could have applied is noise in gene expression. Noise in gene expression is known to be one of the major sources of population heterogeneity in bioprocesses and appears even in stable environments [63, 64]. Noise in gene expression for single‐cell growth and general stress response was only elevated during the transition from the biomass to the product formation phase. Surprisingly, noise in gene expression levels for the general stress response were lower than for the other markers, which was unexpected as *rpoS* expression is known be noisy especially in comparison to the expression of genes that are related to the growth rate [65]. Therefore, this finding needs further investigation in future experiments. Other than that, the cells seemed to employ noise in gene expression in the *nar*‐operon during the product formation phase. However, this strategy did not seem to be sustainable against metabolic stress as after around 75 h, product formation collapsed and cell activity gradually declined. All subpopulations found should be further characterized by proteome analysis after sorting to identify factors that contribute to cell robustness of *E. coli* FUS4 (pF81_kan_). It should generally be mentioned that none of the subpopulations that appeared during the process for L‐phe production was clearly resolved from the main population and permanent. They rather temporary arose in certain process phases and afterwards reverted again. However, due to the well‐mixed conditions provided in the laboratory scale stirred‐tank bioreactor applied in this study this might not be surprising.

## 5 CONCLUDING REMARKS

Overall, application of the 3RP during the L‐phe production process could provide further additional insights to population level physiology. The level of heterogeneity was mostly elevated during the transition between different process phases. This is related to the homogeneous conditions in well‐mixed lab‐scale stirred‐tank bioreactors, in which the cells mostly experience the same ideal conditions and therefore behave equally [16]. Therefore, future studies should aim to induce dynamic conditions, for example, applying a multicompartment system to simulate conditions that would arise during scale‐up [66, 67, 68]. This setup would probably induce higher levels of heterogeneity and thus more permanent subpopulation formation.

Furthermore, investigations regarding the product formation decline appear to be interesting. Here, advancing the strain with another fluorescent protein for monitoring of single‐cell product formation could be interesting. This could be integrated on the plasmid that carries the genes for finalizing L‐phe synthesis so that its expression would be correlated to product formation.

In long‐term perspective, it is desirable to influence the level of population heterogeneity in the L‐phe production process to induce the appearance of process beneficial subpopulations.

## CONFLICT OF INTEREST

The authors have declared no conflicts of interest.

## AUTHOR CONTRIBUTIONS

Manh Dat Hoang planned and conducted the experiments, structured and drafted the manuscript. Dieu Thi Doan supported the data analysis. Marlen Schmidt and Harald Kranz constructed and provided the triple reporter strain. Andreas Kremling contributed to the data analysis. Anna‐Lena Heins is the corresponding author, supported the development of experiments and critically revised the manuscript. All authors gave consent and read and approved the final manuscript.

## Supporting information

Supplementary material
Supplementary material can be found inline in the Supplementary material section at the end of the article.

## Data Availability

The data that support the findings of this study are available from the corresponding author upon reasonable request.
