# Supplementary material for: Application of an Escherichia coli triple reporter strain for at‐line monitoring of single‐cell physiology during L‐phenylalanine production
Source: Eng Life Sci. 2022 Feb 26;23(1):e2100162. doi: 10.1002/elsc.202100162 (PMC9815085; doi:10.1002/elsc.202100162)
Supplement: Supplementary file 1 — Supplementary material Supplementary material can be found inline in the Supplementary material section at the end of the article. [file ELSC-23-e2100162-s001.pdf]

Supplementary material

Application of an *Escherichia coli* triple reporter strain for *at-line* monitoring of single-cell physiology during L-phenylalanine production

Manh Dat Hoang[1]
Dieu Thi Doan[2]
Marlen Schmidt[3]
Harald Kranz[3]
Andreas Kremling[2]
Anna-Lena Heins[1]

[1]Technical University of Munich, TUM School of Engineering and Design, Department of Energy and Process Engineering, Chair of Biochemical Engineering, Garching, Germany
[2]Technical University of Munich, TUM School of Engineering and Design, Department of Energy and Process Engineering, Systems Biotechnology, Garching, Germany
[3]Gen-H Genetic Engineering Heidelberg GmbH, Heidelberg, Germany

Correspondence: Dr. Anna-Lena Heins (anna-lena.heins@tum.de). Technical University of Munich, TUM School of Engineering and Design, Department of Energy and Process Engineering, Chair of Biochemical Engineering, Boltzmannstr. 15, 85748 Garching, Germany.

Table SI: A list of *Escherichia coli* strains which were used for preliminary studies of the fluorescent proteins mTagBFP2, eGFP (as substitute for mEmerald) and CyOFP1. Each E. coli strain harbours one fluorescent protein. The origin laboratories of the strains are listed, as well.

Strain	Fluorescent protein decoded	Origin laboratory
E. coli BL21 (DE3)	mTagBFP2	Stark et al. 2018
E. coli BL21 (DE3)	eGFP	Intern
E. coli DH5α	CyOFP1	Chu et al. 2016
E. coli K-12 (FUS4)	Negative control	Weiner et al. 2014

Figure S1: Comparative fluorescence intensity measurement of mTagBFP2, eGFP and CyOFP1 with BD FACSMelody™ at 448/45 nm (PMT at 328 mV) with 100.000 recorded events for each measurement. Fluorescent proteins were recombinantly expressed by either *Escherichia coli* DH5α or *E. coli* BL21 cells. The negative control displays the autofluorescence of *E. coli* FUS4 cells.

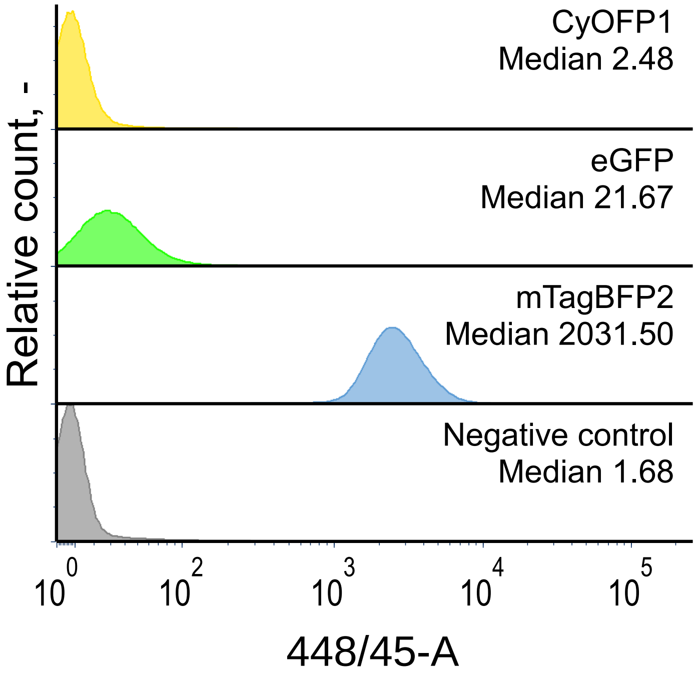

Figure S2: Comparative fluorescence intensity measurement of mTagBFP2, eGFP and CyOFP1 with BD FACSMelody™ at 527/32 nm (PMT at 294 mV) with 100,000 recorded events for each measurement. Fluorescent proteins were recombinantly expressed by either *Escherichia coli* DH5α or *E. coli* BL21 cells. The negative control displays the autofluorescence of *E. coli* FUS4 cells.

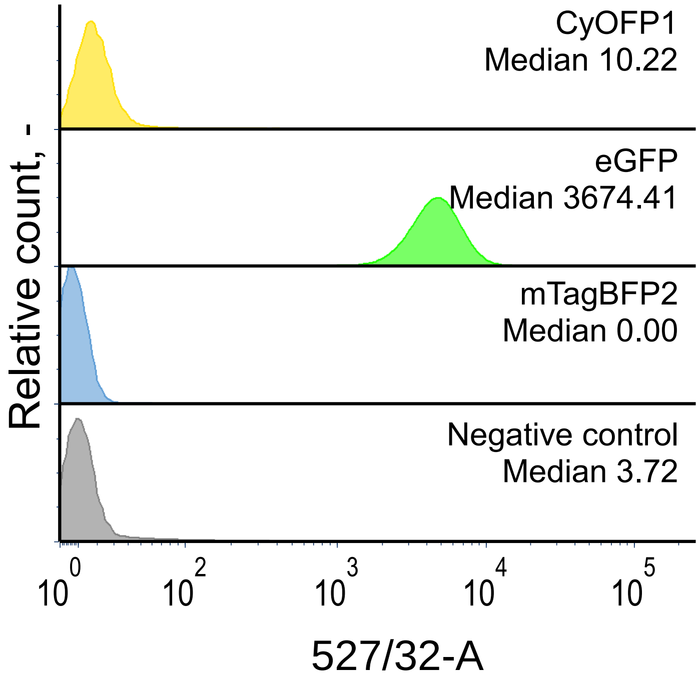

Figure S3: Comparative fluorescence intensity measurement of mTagBFP2, eGFP and CyOFP1 with BD FACSMelody™ at 586/42 nm (PMT at 425 mV) with 100,000 recorded events for each measurement. Fluorescent proteins were recombinantly expressed by either *Escherichia coli* DH5α or *E. coli* BL21 cells. The negative control displays the autofluorescence of *E. coli* FUS4 cells.

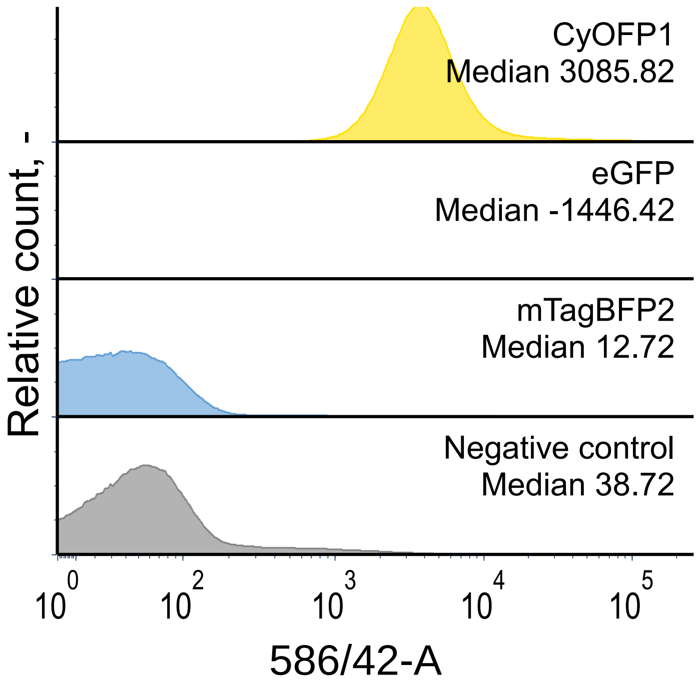

References

Stark et al. (2018). BioBits™ Bright: A fluorescent synthetic biology education kit. *Science Advances*, *4*(8), eaat5107.

Chu et al. (2016). A bright cyan-excitable orange fluorescent protein facilitates dual-emission microscopy and enhances bioluminescence imaging in vivo. *Nature Biotechnology*, *34*(7), 760-767.

Weiner et al. (2014). Carbon storage in recombinant *Escherichia coli* during growth on glycerol and lactic acid. *Biotechnology and Bioengineering*, *111*(12), 2508-2519.
